# Incidence and factors associated with hypoglycemia in newly diagnosed hospitalized children and adolescents with type 1 diabetes mellitus in China

**DOI:** 10.3389/fcdhc.2026.1805855

**Published:** 2026-05-26

**Authors:** Wang Li, Lin Qin, Zhu Gaohui, Chen Lili, Guo Rong, Wu Liping

**Affiliations:** 1 Outpatient Department, Children’s Hospital of Chongqing Medical University, National Clinical Research Center for Children and Adolescents’ Health and Diseases, Ministry of Education Key Laboratory of Child Development and Disorders, Intelligent Application of Big Data in Pediatrics Engineering Research Center of Chongqing Education Commission of China, Chongqing, China; 2 Department of Endocrinology, Children’s Hospital of Chongqing Medical University, Chongqing, China; 3 Chongqing Department of Respiratory Medicine, Affiliated Children’s Hospital of Chongqing Medical University, Chongqing, China; 4 Department of Nursing, Affiliated Children’s Hospital of Chongqing Medical University, School of Nursing, Chongqing Medical University, Chongqing, China; 5 Nursing Office, Jiangxi Hospital, Affiliated Children’s Hospital of Chongqing Medical University, Nanchang, China

**Keywords:** associate factors, children and adolescents, hospitalized, hypoglycemia, type 1 diabetes mellitus

## Abstract

**Objective:**

To investigate the incidence and associated factors of hypoglycemia in hospitalized children and adolescents with newly diagnosed type 1 diabetes mellitus(T1DM) at a pediatric medical center in China.

**Research design and methods:**

This retrospective study included 567 children and adolescents aged <18 years with newly diagnosed T1DM hospitalized between January 1, 2016, and January 1, 2023. Hypoglycemia was defined as blood glucose <3.9 mmol/L (grade 1: 3.0–3.9 mmol/L; grade 2: <3.0 mmol/L).Between-group comparisons were performed using independent samples t-test, χ2-test, Mann-Whitney U-test, and binary logistic regression analysis to identify factors associated with hypoglycemia.

**Results:**

Among 567 patients, The total number of blood glucose measurements during hospitalization was 50,626.Hypoglycemia (<3.9 mmol/L) occurred 2,545 times, with an incidence density of 5.03%.Regarding severity 1,995 episodes of grade 1 hypoglycemia occurred(incidence density 3.94%), and 550 episodes of grade 2 hypoglycemia occurred(incidence density 1.08%).In terms of frequency, 81.66% (463/567) of patients had at least 1 hypoglycemia episode during hospitalization. Among those, 15.87% (90/567) had one episode, 12.52% (71/567) had two episodes, and 53.26% (302/567) had three or more episodes. The recurrence rate (≥2 episodes) was 65.78%(373/567 ).Regarding timing, grade 1 hypoglycemia occurred most frequently at night (00:00–06:00), while the highest event density for grade 2 was observed in the pre-lunch period (09:00–12:00). Multifactorial logistic regression analysis showed that age 7–12 years(OR = 0.421,95%CI=0.244-0.727,p*=*0.002),longer hospitalization days (OR=1.346,95%CI=(1.217-1.488, p<0.001), use of multiple daily injections (MDI) + continuous subcutaneous insulin infusion (CSII) regimen (OR = 3.232, 95%CI=1.399-7.469, p=0.006), fasting C-peptide (OR = 0.200, 95%CI=0.076-0.525, p*=*0.001), and fasting insulin (OR = 0.938, 95%CI=0.890-0.988, p=0.017) were independently associated with hypoglycemia.

**Conclusions:**

Hypoglycemia is common among newly diagnosed hospitalized children and adolescents with T1DM in China, particularly at night for grade 1 events. Age, hospitalization days, insulin regimen, fasting C-peptide, and fasting insulin were associated with hypoglycemia. These associations, particularly for insulin regimen, should be interpreted considering potential confounding by indication due to the observational study design. These findings may inform targeted prevention strategies.

## 1 Introduction

Type 1 diabetes mellitus (T1DM) is an autoimmune disorder characterized by the destruction of pancreatic β-cells, leading to absolute insulin deficiency subsequent disorders of glucose, fat, and protein metabolism (1) T1DM is the most prevalent chronic endocrine disorder among the pediatric population (2). Globally, an estimated1.2 million children and adolescents have T1DM, with 180,000 new cases diagnosed annually (3). Intensive insulin therapy is the mainstay of clinical treatment for T1DM.Although it helps achieve glycemic targets and prevent chronic complications associated with diabetes, it increases the risk of hypoglycemia (4).

Recurrent hypoglycemia episodes have been shown to be associated with cognitive impairment (4, 5), increased risk of cardiovascular events and all-cause mortality (6). On a psychological level, recurrent hypoglycemia can trigger fear of hypoglycemia in both the child and their family members (7). This fear may create a vicious cycle in blood glucose management, increasing the risk of diabetes complications and ultimately leading to a decline in the quality of life. Studies have shown that frequent hypoglycemia during hospitalization is associated with prolonged hospital and poorer glycemic control after discharge, which increases healthcare resources and the family care burden (8).

According to the Diabetes UK Combined Treatment Group Flow Survey, the prevalence of grade 2 hypoglycemia (<3.0 mmol/L) in patients hospitalized with T1DM in the UK was 27% in 2019 (9), compared with 30.4% in 2017 and 28.0% in 2010, suggesting that the incidence has not significantly decreased over the decade despite advances in medical technology (10) Large-scale epidemiological surveys on hypoglycemia in hospitalized T1DM population are lacking in China. One study reported that the incidence of hypoglycemia in children and adolescents hospitalized in Guangzhou was 61.85% during 2015-2016 (11), and 60.9% during 2017-2019 (12). Another study in Henan region reported an incidence of 50.49% (13). The recurrence of hypoglycemia is also noteworthy a study of 1,7000 children and adolescents worldwide reported that 45% of hospitalized patients experienced recurrence of hypoglycemia (14), while another study reported a rate of 66.0% (15). Hypoglycemia has become a prominent problem in the treatment of children and adolescents with T1DM and an important limiting factor in achieving optimal glycemic control.

Our hospital is one of the major treatment centers for children and adolescents with diabetes in China, serving patients mainly from the southwestern region of China and across the country. In this study, we systematically review the current status of hypoglycemia and its influencing factors in children and adolescents with T1DM in our hospital, aiming to provide epidemiological evidence for hypoglycemia prevention and treatment in this population.

## 2 Research design and methods

### 2.1 Study population

**Figure 1:**
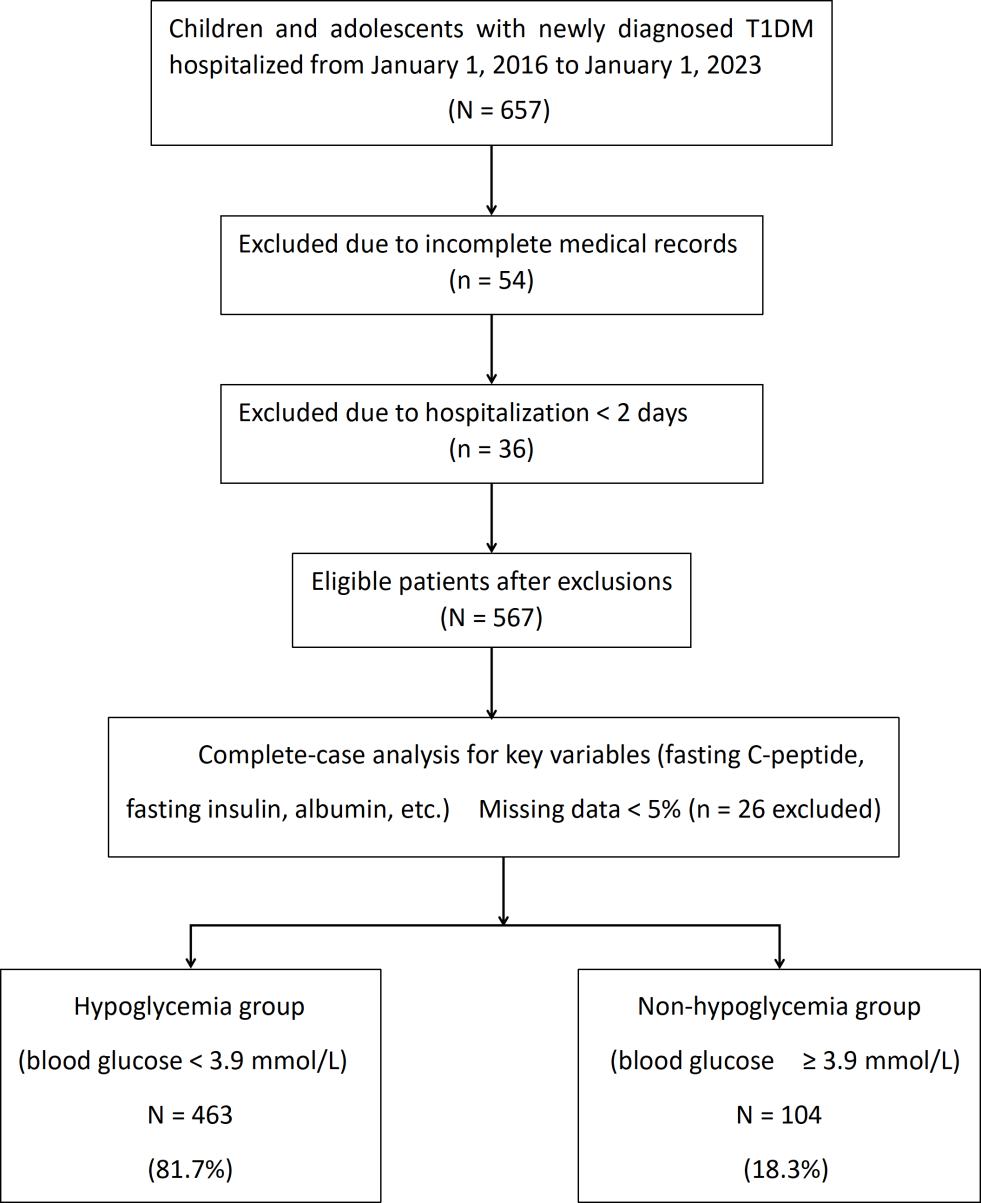
Study flow diagram of participant selection.

**Figure 1** presents a flowchart of patient selection A total of 657 children with newly diagnosed T1DM were initially identified; 90 were excluded due to incomplete medical records (n=54) or hospitalization <2 days (n=36), leaving 567 children for analysis. The study was approved by the Ethics Committee of the Affiliated Children’s Hospital of Chongqing Medical University under the (approval number [2021]: Nianlun Audit [Research] No. 356). Inclusion criteria were: (1) children and adolescents newly diagnosed with T1DM according to the 1999 World Health Organization (WHO) diabetes mellitus diagnostic criteria (16) (2) aged <18 years; (3) hospitalization > 1 day with >8 blood glucose measurements. Exclusion criteria: those with missing or incomplete case data.

### 2.2 Study indicators

#### 2.2.1 General information

Data collected included: gender, age, date of birth, weight, height, place of residence, caregiver literacy and education level, health insurance status, disease of duration, history of T1DM and other comorbidities. presence of diabetic ketoacidosis (DKA) at admission, insulin dose, type, and injection method.

#### 2.2.2 Laboratory tests

Laboratory measurements included glycated hemoglobin (hemoglobinHbA1C), blood glucose monitoring values, white blood cells, red blood cells, lymphocytes, neutrophils, platelets, albumin, hemoglobin, total protein, glutamate aminotransferase, aspartate aminotransferase, and other relevant indicators. The following composite indices were calculated:

1. Prognostic Nutritional Index (PNI) = albumin value + 0.005 x lymphocyte count
2. Neutrophil-albumin Ratio (NAR) = neutrophil count/albumin
3. Platelet-Albumin Ratio (PAR) = platelet count/albumin

These indices have been used to assess nutritional and immune status in various clinical contexts (17–19).

#### 2.2.3 Treatment regimen classification

The primary insulin regimen used in the Department of Endocrinology during hospitalization was recorded for classification. Patients transferred from other department were not included. Patients treated with Continuous Subcutaneous Insulin Infusion (CSII) were classified as the “CSII group”, those treated with multiple daily injections (MDI) as “MDI group”, and those using intravenous insulin as the “ Intravenous group”. For patients who received multiple regimens during the same,they were grouped according to the combination of regimens used.(e.g., patients first treated with MDI and then changed to MDI+CSII, were classified as MDI+CSII group). The analysis of associations between insulin regimen and hypoglycemia should be interpreted with caution due to potential confounding by indication.

#### 2.2.4 Hypoglycemia definition and grouping

According to the American Diabetes Association (ADA) Criteria (20) hypoglycemia was defined as blood glucose concentrations <3.9 mmol/L.Grade 1 hypoglycemia was defined as blood glucose 3.0–3.9 mmol/L, representing the threshold for clinical intervention. Grade 2 hypoglycemia was defined as blood glucose<3.0 mmol/L representing the threshold for onset of neurological symptoms requiring immediate action. Grade 3 hypoglycemia (severe events characterized by altered mental and/or physical status requiring assistance) was not separately analyzed due to the young age of many patients and reliance on caregiver reporting. Patients with one or more episodes of blood glucose <3.9 mmol/L during hospitalization were considered the hypoglycemic group, and the remainder as the non-hypoglycemia group.

#### 2.2.5 Specimen collection and testing

All blood glucose measurements were performed using the same brand of glucose meter, which underwent quality control daily using specialized quality control solution by trained nurses. Plasma glucose values were compared biochemically on an annual basis All nurses performing point-of-care testing (POCT) were certified through in-hospital training. Continuous glucose monitoring (CGM) was not used during the study period in our center.

### 2.3 Statistical analysis

Statistical analyses were performed using SAS 9.4. Count data were described as numbers and percentages, the chi-square test was used for comparison between groups. Measurement information of normal distribution was described by mean plus or minus standard deviation, and t-test was used for comparison between groups. Measures with skewed distribution were described by median and interquartile spacing, and comparisons between groups were made using the Mann-Whitney U test. Categorical information was described using the number of cases and percentages, and comparisons between groups were made using the chi-square test, with *post hoc* two-by-two comparisons adjusted for P values using the Bonferroni method.

Variables with p*<*0.10 in the univariate analysis were included in the multivariate logistic regression model. Stepwise regression was applied for variable selection, with entry criterion p<0.05 and removal criterion p≥0.05. A hierarchical modeling approach was used: separate models were first constructed for each category of indicators (general data, glucose control regimen, nutritional/infection indicators), and variables with p < 0.05 from each model were then included in the final full model. Two-sided p < 0.05 was considered statistically significant.

### 2.4 Study population characteristics

A total of 567 children and adolescents with newly diagnosed T1DM were enrolled in the study. Among them, 276 cases (48.7%) were age0–6 years, 260 cases (45.8%) were aged 7–12 years, and 31 cases (5.5%) were aged 13–18 years. The hypoglycemia group (≥1 episode) comprised 463 cases (81.7%), including 219 males and 244 females; the non - hypoglycemia group consisted of 104 cases (18.3%), including 45 males and 59 females.

### 2.5 Blood glucose monitoring and hypoglycemia incidence

The total number of blood glucose monitoring during hospitalization was 50,626, Hypoglycemia (<3.9 mmol/L) occurred 2545 times, with an incidence density of 5.03%.Grade 1 hypoglycemia (3.0-3.9 mmol/L) occurred 1,995 times (3.94%), and grade 2 hypoglycemia (<3.0 mmol/L) occurred 550 times (1.08%). Of the 567 children, 463 (81.66%) had at least one episode of hypoglycemia. Among these, 90 (15.87%) had one episode, 71 (12.52%) had two episodes, and 302 (53.26%) had three or more episodes, yielding a recurrence rate (≥2 episodes among all patients) of 65.78%.

Regarding timing of hypoglycemia: for grade 1, the highest proportion of episodes occurred at night (00:00–06:00, 29.59% of grade 1 episodes). For grade 2, the highest event density (episodes per total measurements in that time period) was observed in the pre-lunch period (09:00–12:00, 1.81%), not at night (0.99%). The density of hypoglycemia at different time periods is shown in **Table 1**.

**Table 1:** Density of hypoglycemia at different times in hospitalized children with T1DM [times (%,%).

Time period	Hypoglycemia 1			Hypoglycemia 2		
	Cases (%)	Number of hypoglycemic episodes	Density of hypoglycemic occurrence	Cases (%)	Number of hypoglycemic episodes	Density of hypoglycemic occurrence
Night (0:00~6:00)	321 (29.59%)	739	5.60%	96 (23.24%)	130	0.99%
Before breakfast (6:00~7:00)	3 (0.28%)	3	3.16%	0 (0.00%)	0	0.00%
After breakfast (7:00~9:00)	65 (5.99%)	75	3.96%	22 (5.33%)	25	1.32%
Before lunch (9:00~12:00)	244 (22.49%)	468	4.40%	124 (30.02%)	192	1.81%
After lunch (12:00~14:30)	138 (12.72%)	212	3.99%	46 (11.14%)	50	0.94%
Before dinner (14:30~17:00)	37 (3.41%)	45	2.59%	18 (4.36%)	20	1.15%
After dinner (17:00~19:30)	140 (12.90%)	225	2.41%	62 (15.01%)	73	0.78%
Bedtime (19:30~24:00)	137 (12.63%)	228	2.70%	45 (10.90%)	60	0.71%

### 2.6 Univariate analysis

Univariate analysis showed statistically significant differences (p < 0.05) between the hypoglycemia and non-hypoglycemia groups in age, hospitalization days, fasting C-peptide, fasting insulin, average daily insulin per body weight, albumin, hemoglobin, total protein, lymphocytes, PNI score, and PAR.,Specifically, patients in the hypoglycemia group had longer hospitalization days (11 vs 8.5, days), higher lymphocyte counts (2.78 vs 2.59), and higher PAR counts (7.02 vs 6.35, 10^9^/g) compared to the non-hypoglycemia group. Conversely, the hypoglycemia group had lower fasting C-peptide (0.21 vs 0.35, ng/ml), lower fasting insulin (3.00 vs 3.80, mIU/l), lower daily average insulin/body weight (0.6 vs 0.76, U/kg), lower albumin (41.59 vs 43.20, g/L), lower hemoglobin (126.66 vs 132.77, g/L), lower total protein (65.90 vs 68.33, g/L), and lower PNI scores (41.61 vs 43.21).The prevalence of hypoglycemia was highest in children aged 0–6 years(90.94%), significantly higher than in children aged 7–12 years(72.69%)and 13–18 years(74.19%).

No statistically significant difference between groups in gender, DKA at admission, comorbid renal disease, comorbid liver disease, comorbid eye disease, comorbid dyslipidemia, comorbid abnormalities of thyroid function, type of insulin used, HbA1c, neutrophil count, platelets count, and NAR, P>0.05. See **Table 2** for details.

**Table 2:** Univariate analysis of hypoglycemic events in children hospitalized with T1DM (n=567).

Considerations	No hypoglycemia group (n=104)	Hypoglycemic group (n=463)	χ2/t/Z value	P-value
General information				
Age (years, [cases (%)])			30.990	<0.001
0~6	25 (9.06)	251 (90.94)		
7~12	71 (27.31)	189 (72.69) a		
13~18	8 (25.81)	23 (74.19) a		
Sex [cases (%)]			0.555	0.456
male	45 (17.05)	219 (82.95)		
women	59 (19.47)	244 (80.53)		
Admission DKA [cases (%)]			1.302	0.729
clogged	28 (17.28)	134 (82.72)		
unimportant	50 (19.01)	213 (80.99)		
center	13 (22.41)	45 (77.59)		
repetition	13 (15.48)	71 (84.52)		
Days of hospitalization (days, [M(P25,P75)])	8.5 (7, 11)	11 (9, 13)	-6.191	<0.001
Comorbidities [cases (%)]				
Combined kidney disease			2.673	0.102
clogged	102 (18.96)	436 (81.04)		
be	2 (6.90)	27 (93.10)		
Combined liver disease			0.144	0.705
clogged	98 (18.11)	443 (81.89)		
be	6 (23.08)	20 (76.92)		
Combined eye diseases			2.018	0.155
clogged	102 (18.09)	462 (81.91)		
be	2 (66.67)	1 (33.33)		
Combined dyslipidemia			0.211	0.646
clogged	89 (18.05)	404 (81.95)		
be	15 (20.27)	59 (79.73)		
Combined Thyroid Abnormalities			1.729	0.189
clogged	94 (17.77)	435 (82.23)		
be	10 (26.32)	28 (73.68)		
Glycemic control program and related indicators [M(P25,P75)]				
Fasting C-peptide (ng/ml)	0.35 (0.2, 0.61)	0.21 (0.11, 0.36)	5.041	<0.001
Fasting insulin (mIU/l)	3.8 (1.95, 6.7)	3 (1.3, 5.42)	2.711	0.007
Average daily insulin/body weight (U/kg, [M(P25,P75)])	0.76 (0.55, 0.98)	0.6 (0.43, 0.83)	3.830	<0.001
Insulin injection regimen [cases (%)]			7.075	0.070
MDI	23 (19.83)	93 (80.17)		
MDI+CSII	10 (11.49)	77 (88.51)		
MDI + IV	39 (23.93)	124 (76.07)		
MDI + CSII + IV	32 (15.92)	169 (84.08)		
Type of insulin used [cases (%)]			1.222	0.543
1 type	23 (19.83)	93 (80.17)		
2 types	49 (19.60)	201 (80.40)		
3 types	32 (15.92)	169 (84.08)		
HbA1c (%, [example (%)])			/	0.869
<7	0 (0.00)	1(100.00)		
7~10	7 (16.28)	36 (83.72)		
>10	97 (18.55)	426 (81.45)		
Nutritional indicators ( )				
Albumin (g/L)	43.2±4.83	41.59±5.43	2.777	0.006
Hemoglobin (g/L)	132.77±15.32	126.66±15.02	3.737	<0.001
Total protein (g/L)	68.33±7.66	65.9±7.42	2.989	0.003
Infection indicators [M(P25,P75)]				
lymphocyte	2.59 (2.12, 3.08)	2.78 (1.93, 4.06)	-2.039	0.041
Neutrophils (109 /L)	3.54 (2.86, 5.26)	3.69 (2.62, 6.04)	-0.316	0.752
Platelets (109 /L)	274 (233.5, 333)	286.5 (235, 353)	-1.169	0.242
Nutritional Infection Composite Indicator				
PNI score ( )	43.21±4.83	41.61±5.43	2.772	0.006
NAR (109 /g) [M(P25,P75)]	0.08 (0.06, 0.12)	0.09 (0.06, 0.16)	-0.824	0.410
PAR (109 /g) [M(P25,P75)]	6.35 (5.54, 7.63)	7.02 (5.63, 8.49)	-2.325	0.020

MDI is multiple daily subcutaneous injections; CSII is continuous subcutaneous insulin infusion; HbA1c is glycosylated hemoglobin. Post hoc two-by-two comparisons were made using the Bonferroni method adjusted for P values,a p<0.05 when compared with 0-6 years of age; PNI is Prognostic Nutritional Index; NAR is Neutrophil Value/Albumin Value; PAR is Platelet/Albumin Value.

### 2.7 Multifactorial logistic regression analysis

**Tables 3A, B** presents the results of the multivariable logistic regression model. A hierarchical modeling approach was used: Model 1 included general characteristics, Model 2 included glucose control regimen and related indicators, and Model 3 included nutritional, inflammatory, and composite indicators. The final full model included all variables with p < 0.05 from Models 1–3.

**Table 3A:** Assignment of independent variables in multifactor logistic regression model (n=567).

Independent variable	Variable assignment
Age (years)	Dummy variables are set with reference to "0~6", 7~12=(Z1=1, Z2=0); 13~18=(Z1=0, Z2=1)
Days of hospitalization (days)	original value
Fasting C-peptide (ng/ml)	original value
Fasting insulin (mIU/l)	original value
Average daily insulin/body weight (U/kg)	original value
Insulin injection program	Dummy variables were set up with reference to "MDI + IV", MDI = (Z1=1, Z2=0, Z3=0); MDI + CSII = (Z1=0, Z2=1, Z3=0); MDI + CSII + IV = (Z1=0, Z2=0, Z3=1)
Albumin (g/L)	original value
Hemoglobin (g/L)	original value
Total protein (g/L)	original value
lymphocyte	original value
PNI score	original value
PAR (109 /g)	original value

PNI is Prognostic Nutritional Index; PAR is platelet/albumin value.

**Table 3B:** Multifactorial logistic regression model of hypoglycemia in children with newly diagnosed T1DM.

Independent variable	Beta value	Standardization ratio	Standard error	χ2 value	P-value	OR (95% CI)
Model 1 general information						
Age (years)						
0~6						1.0 (reference)
7~12	-1.244	-0.342	0.260	22.859	<0.001	0.288 (0.173, 0.480)
13~18	-1.189	-0.149	0.474	6.293	0.012	0.305 (0.120, 0.771)
Days of hospitalization (days)	0.224	0.489	0.042	28.934	<0.001	1.251 (1.153, 1.357)
Model 2 sugar control program and related indicators						
Fasting C-peptide (ng/ml)	-1.876	-0.259	0.419	20.060	<0.001	0.153 (0.067, 0.348)
Fasting insulin (mIU/l)	-0.059	-0.141	0.025	5.730	0.017	0.943 (0.899, 0.989)
Insulin injection program						
MDI	0.146	0.032	0.307	0.224	0.636	1.157 (0.633, 2.112)
MDI+CSII	0.904	0.180	0.397	5.175	0.023	2.468 (1.133, 5.377)
MDI + IV						1.0 (reference)
MDI + CSII + IV	0.357	0.094	0.285	1.568	0.211	1.429 (0.817, 2.498)
Model 3 nutrition, infections and their composite indicators						
Hemoglobin (g/L)	-0.025	-0.207	0.007	11.669	0.001	0.976 (0.962, 0.990)
PAR (10^9^ /g)	0.109	0.141	0.052	4.320	0.038	1.115 (1.006, 1.235)
Full model						
a constant (math.)	-0.466	-	0.570	0.669	0.413	
Age (years)						
0~6						1.0 (reference)
7~12	-0.865	-0.238	0.278	9.647	0.002	0.421 (0.244, 0.727)
13~18	-0.202	-0.025	0.547	0.137	0.711	0.817 (0.280, 2.386)
Days of hospitalization (days)	0.297	0.650	0.051	33.575	<0.001	1.346 (1.217, 1.488)
Fasting C-peptide (ng/ml)	-1.609	-0.222	0.492	10.679	0.001	0.200 (0.076, 0.525)
Fasting insulin (mIU/l)	-0.064	-0.154	0.027	5.747	0.017	0.938 (0.890, 0.988)
Insulin injection program						
MDI	0.928	0.207	0.359	6.664	0.010	2.529 (1.250, 5.115)
MDI+CSII	1.173	0.233	0.427	7.534	0.006	3.232 (1.399, 7.469)
MDI + IV						1.0 (reference)
MDI + CSII + IV	0.130	0.034	0.305	0.183	0.669	1.139 (0.627, 2.070)

PAR is platelet/albumin value; MDI is multiple daily subcutaneous injection; CSII is continuous subcutaneous insulin infusion.

The final full model showed the following independent associations with hypoglycemia:

Aged 7–12 years was associated with lower odds of hypoglycemia compared to 0–6 years (OR = 0.421,95%CI=0.244~0.727,p=0.002). Age13–18 years was not significantly associated with hypoglycemia (OR = 0.817,95%CI:0.280-2.386,p= 0.711).

Longer hospitalization days were associated with higher odds of hypoglycemia (OR = 1.346 per day increase, 95%CI:1.217-1.488, p<0.001).

Higher fasting C-peptide(OR = 0.200,95%CI:0.076-0.525,p =0.001) and higher fasting insulin(OR = 0.938,95%CI:0.890-0.988, p=0.017) were associated with lower odds of hypoglycemia.

Insulin regimen was associated with hypoglycemia. Compared to the MDI + IV group, the MDI alone group (OR = 2.529,95%CI:1.250-5.115, p=0.010) and the MDI + CSII group (OR = 3.232,95%CI:1.399-7.469,p=0.006) had higher odds of hypoglycemia. The MDI + CSII + IV group was not significantly associated with hypoglycemia (OR = 1.139,95%CI:0.627–2.070,p=0.669).

## 3 Discussion

In this study, the incidence of any hypoglycemia (<3.9 mmol/L) in newly diagnosed hospitalized children and adolescents with T1DM was 81.66%, which was higher than the rates reported from Guangzhou(61.85% in 2015-2016, 60.9% in 2017-2019) (13) and Henan (50.49% in 2017-2019) (14). Although direct cross-country comparisons should be made with caution due to differences in healthcare systems and monitoring protocols, these findings suggest a substantial burden. Consistent with previous studies (15–17), the highest number of grade 1 hypoglycemia episodes occurred at night. However, the highest density of grade 2 hypoglycemia was observed in the pre-lunch period, suggesting that more severe hypoglycemic events may follow morning insulin dosing and activity patterns. The recurrence rate(≥2 episodes) in this study was 65.78%, indicating that patients who experienced one hypoglycemia episode were highly susceptible to recurrence of hypoglycemia. Several studies have shown that CSII and continuous glucose monitoring can reduce the incidence of hypoglycemia in children with T1DM (18–20). However, because our Center in is located in an inland area of China, with an intermediate regional economy and medical resources, wider clinical adoption of these technologies will require time.

Hypoglycemia occurrence in newly diagnosed hospitalized children and adolescents with T1DM was associated with multiple factors. In this study, the odds of hypoglycemia in children aged 7 to 12 years were 0.421 times higher than those of children aged 0 to 6 years (OR = 0.421). A systematic review of prediction models for hypoglycemia risk showed that 10 of 16 published modelings used age as a predictor (21), suggests age is an important factor associated with hypoglycemia risk in both T1DM and T2DM.Children aged group 0–6 years are highly susceptible to hypoglycemia due to inconsistent eating patterns relative to insulin administration and their inability to self-manage or seek help. As children reach adolescence(13-18 years),hypoglycemia incidence may enters another peak due to sex hormone changes and emotional instability (22). Therefore, anticipatory guidance and corresponding health education programs for adolescent patients and caregivers are necessary (23).

Each additional day of hospitalization was associated with 1.346 times higher fodds of hypoglycemia(OR = 1.346). Zuo (24) et al. also included hospitalization duration (OR = 2.052) as a predictor in their modeling for hypoglycemia in T2DM patients. It is plausible that longer hospital stays increase the opportunity for hypoglycemia detection. Additionally, patients with prolonged hospitalizations may have more severe disease, greater glucose variability, and more comorbidities, which could jointly increase hypoglycemia risk (25).

Most studies have shown that CSII is associated with lower hypoglycemia risk compared to MDI (26, 27). However, approximately 40% of children and adolescents with T1DM are with DKA requiring continuous low-dose intravenous insulin before changing to MDI or CSII (28) In our observational analysis, the MDI+CSII regimen was associated with higher odds of hypoglycemia compared to MDI+IV (OR = 3.232). This association should not be interpreted as causal. It is more likely explained by confounding by indication: patients who required CSII in addition to MDI may have had more brittle diabetes or poorer initial glycemic control, making them inherently more prone to glucose fluctuations and hypoglycemia regardless of the regimen (29–31). For this group, careful insulin dose adjustment and close glucose monitoring during regimen transitions are essential.

Consistent with the DPT-1 study (32), which incorporated age, glucose, and C-peptide to predict T1DM risk, our study found that lower fasting C-peptide (OR = 0.200) and lower fasting insulin (OR = 0.938) were associated with higher odds of hypoglycemia. These findings suggest that more severe β-cell destruction (reflected by lower fasting C-peptide/insulin) may create “brittle” glucose control, where even small mismatches between exogenous insulin and glucose intake produce wide glucose fluctuations, increasing hypoglycemia risk. Similar results were obtained in clinical analyses of fulminant T1DM in both children (33) and adults (34).

Insulin overdose is a traditional concern for hypoglycemia (35). However, in this study, the average daily insulin dose/body weight in the hypoglycemic group was lower than the non-hypoglycemic group, suggesting that the higher insulin dose was not associated with higher hypoglycemia incidence. This counterintuitive finding may reflect that patients with more resistant hyperglycemia (requiring higher doses) had some residual β-cell function, while those with severe deficiency required lower doses but experienced greater glucose lability. Further studies are needed to clarify this relationship.

HbA1C reflects blood glucose over the preceding three months. Most studies have confirmed that lower HbA1c is associated with an increased hypoglycemia risk in T1DM (18–20, 36–38). However, a global multicenter HAT study showed no such association (3), and our study also showed that there was no statistically significant association between admission HbA1C and inpatient hypoglycemia. Therefore, the values of HbA1C for predicting inpatient hypoglycemia requires further investigation A history of out-of-hospital hypoglycemia is an important factor that we could not assess due to study design (39).

### 3.1 Limitations

This study has several limitations. First, the retrospective, single-center design limits generalizability and introduces potential selection bias. Second, treatment regimen was not randomized; therefore, confounding by indication cannot be excluded. Third, hospitalization days reflect both biological risk and opportunity for detection; although we performed a sensitivity analysis using event density, residual confounding remains possible. Fourth, blood glucose monitoring frequency, although standardized per protocol, may have varied between patients. Fifth, we lacked data on out-of-hospital hypoglycemia history and fear of hypoglycemia, which are important clinical factors. Finally, comparisons with historical studies using different hypoglycemia thresholds should be interpreted cautiously.

## 4 Conclusions

Hypoglycemia is common among newly diagnosed hospitalized children and adolescents with T1DM in China, particularly at night for grade 1 events. Age, hospitalization days, insulin regimen, fasting C-peptide, and fasting insulin are associated with hypoglycemia. These findings may help clinicians identify high-risk children and adolescents and design targeted prevention strategies. Future prospective multicenter studies with standardized protocols and longer follow-up are needed to confirm these associations and explore causal relationships.

## Data Availability

The original contributions presented in the study are included in the article/supplementary material. Further inquiries can be directed to the corresponding author.
